# Leptin hormone and its effectiveness in reproduction, metabolism, immunity, diabetes, hopes and ambitions

**DOI:** 10.25122/jml-2021-0153

**Published:** 2021

**Authors:** Hany Akeel Al-hussaniy, Ali Hikmate Alburghaif, Meena Akeel Naji

**Affiliations:** 1. Department of Pharmacy, Al-Karama Hospital, Baghdad, Iraq; 2. Department of Pharmacy, Ashur University College, Baghdad, Iraq; 3. Department of Family Medicine, University of Baghdad, Baghdad, Iraq

**Keywords:** leptin analog, Metreleptin, endocrine hormone, reproduction, leptin mutations

## Abstract

Leptin is a hormone derived from adipose tissue and the small intestine, mainly in enterocytes; it helps regulate the energy balance by suppressing hunger, resulting in decreased fat mass in adipocytes. Leptin has specific receptors in the ventromedial and arcuate nuclei and other parts of the hypothalamus and the feeding center in the ventral tegmental area. It also plays a role in regulatory aspects other than fat cells, such as obesity, which is linked to a loss of sensitivity of leptin receptors, resulting in an inability to produce satiety and an increase in food intake. Moreover, leptin plays a part in lactation, bone density, the immune system, diabetes treatments, and hypertriglyceridemia. The latest studies in leptin suggest that an analog of leptin may treat DM and hypertriglyceridemia. Further research should be conducted on the effectiveness of leptin on other related diseases.

## Introduction

Leptin is derived from the Greek (*leptons*) from a word meaning *thin*. The leptin hormone is derived from adipose tissue, suggesting that adipose tissue is an endocrine gland, not merely inert fat storage as previously thought [1, 2]. Furthermore, leptin acts as an energy regulator in the brain used to induce anorexic factors and suppress appetite factors, reduce intake, and increase energy expenditure [3]. However, studies have shown that low leptin levels can increase food absorption and suppress energy expenditure; in contrast, increased leptin levels can suppress appetite and increase energy consumption [4]. About 25 years after the discovery of leptin, one comes to mind about the effectiveness of leptin and its use to treat many diseases, both leptin mimics as in obesity and leptin-blockers as in tumors. In this article we discuss the most important topics and the relationship of leptin to immunity, cortisone, and diabetes, in addition to pregnancy, tumors, and others.

## Material and Methods

We conducted a review by searching the Google Scholar, PubMed, and Directory Open access Journal databases for relevant information using keywords such as “leptin, leptin analog, leptin and immunity, resistance to leptin, leptin and corticosteroid, resistance to leptin, and leptin and cancer”. From the articles reviewed, we excluded those on leptin gene polymorphism or those that provided irrelevant information on the leptin effect.

## RESULTS

### Role of leptin in hormone regulation

Leptin has a role in diet-related hormone regulation. However, it is also affected by energy status, sex hormones (e.g., leptin synthesis can be inhibited by thyroid ketone, while estrogen and progesterone promote its synthesis), and the level of various anti-inflammatory mediators [5].

### The effect of leptin and receptors

Leptin hormones interact with a receptor in the hypothalamus, suppressing hunger and stimulating satiety; this is done by interacting with several receptors such as neuropeptide Y (a hunger promoter) [6], as well as anandamide, which is another hunger promoter shared with tetrahydrocannabinol, which binds to the same receptors [7]. Moreover, it increases the synthesis of α-Melanocyte-stimulating hormone (α-MSH), which suppresses hunger; such appetite suppression is long-term. However, it can influence the quick suppression of hunger induced by cholecystokinin (CCK) and the more extended appetite reduction caused by Peptide YY (PYY3-36) [8]. Leptin binds to six types of receptors controlled by the LEPR gene [9]. Once leptin binds to its Ob-Rb receptor, it activates stat3, which is phosphorylated and carries the signal to the nucleus, causing changes in gene expression, one of the main effects being down-regulation and a decrease in the number of endocannabinoid receptors, which is associated with increased hunger [10].

### Resistance to leptin

The lack of leptin receptors or leptin itself leads to uncontrolled hunger, resulting in obesity [11]. Leptin concentrations are increased in hypertensive patients, but high endogenous (and exogenous) leptins do not normalize the weight of obese patients, indicating that there is the possibility of leptin resistance, which has been proposed internationally [12]. Furthermore, studies have found a correlation between serum leptin levels and expression levels of Testosterone, estradiol (E2), follicle-stimulating hormone (FSH), and Aromatase (P450)arom in women with hyperandrogenism and Polycystic ovary syndrome (PCOS) [13]. While in vitro experiments have confirmed a negative correlation between thyroxine and leptin, there is still no conclusion in the human body [14]. The existence of leptin is the basis for the exertion of the growth hormone’s physiological effects, but the growth hormone itself does not appear to have a direct effect on leptin [15].

### Leptin and corticosteroids

Fertility Gamma aminobutyric acid (GABA) neurons are necessary for the male leptin metabolism pathway, but they are unnecessary for the leptin reproductive pathway for the maturation and maintenance of the reproductive system [16]. Glucocorticoid GC inhibits the male leptin metabolism-signaling pathway in the center. The effect of GC on leptin in the center is necessary for female fertility [17]. A high level of leptin may be a key pathological characteristic of hyperandrogenic PCOS [18], although aerobic exercise can reduce the level of PCOS leptins by affecting the hypothalamus-pituitary-ovarian axis (HPOA) and improving high androgen. Hyperandrogenemia and sex hormone disorder in PCOS cannot yet be completely improved [13]. The specific mechanism remains to be further studied. Under starvation conditions, GC and leptin independently regulate metabolism and the hypothalamus-pituitary-ovarian axis. For males under stress, GC may not directly act on leptin when regulating the reproductive axis, or, at least, it has a minor effect [19].

Researchers have noted only mild hyperglycemia after treating young patients with different autoimmune diseases with high-dose glucocorticoids (1–2 mg/kg/day prednisone or equivalent methylprednisolone), but early morning hypoglycemia events are not uncommon [20]. Long-term, low-dose glucocorticoid maintenance therapy has little effect on blood glucose in young patients. These studies provide new ideas for exploring pathways in the field of neuroendocrine and blood glucose management [20].

### Leptin and the immune system

In recent years, studies have found that leptin can also widely regulate various immune cells and plays an important role in maintaining the body’s innate immunity and adaptive immune function effects. When significantly elevated, these patients are more susceptible to type 2 diabetes, degenerative diseases, cardiovascular diseases, and autoimmune diseases. However, a decrease in leptin levels (such as in undernourished individuals) can lead to a risk of infection [21, 22].

### The effect of leptin on adaptive immunity

**Figure 1.**
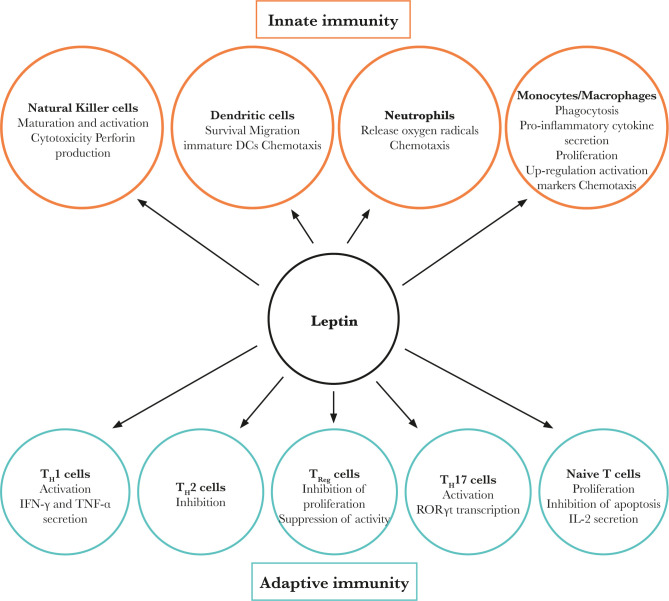
Effect of leptin on innate and adaptive immunity.

In the cell-mediated and humoral immune formula, there are many receptors on the surface [23]. Similarly, infection-related mortality in children is more common in people with congenital leptin insufficiency [24, 25]. In short, leptin promotes T cell activation, proliferation, and cytokine production [26]. Obese mice lacking leptin receptor show thymus atrophy and T cell lymph decrease in cells; leptin facilitates T cells to pass the JAK-STAT signaling pathway signaling pathway, which produces cytokines; leptin can also regulate CD4+ (Figure 1) [25].

### Leptin and cancer studies

Studies conducted on leptin have shown that it may play a role in the occurrence and development of breast cancer [27]. The expression of leptin and its receptor can be used as an indicator of the diagnosis or prognosis of breast cancer [28]. Furthermore, in one study, the leptin levels of the advanced tumor group were higher than that of the early group. Several studies illustrated that leptin might promote the invasion and distant metastasis of colorectal cancer. In addition, leptin may play an important role in the relationship between obesity and colorectal cancer [29]. Recently, several studies have been conducted to study the efficacy of leptin with some anticancer drugs such as doxorubicin [30], which treat a wide range of cancers [31].

### T cell polarization

For Th1 and Th2 cells, leptin enables T helper cytokine production. It has the most important role in body immunity. Research has found that leptin is Th17 necessary for lymphocyte differentiation; in addition, leptin and thymocytes from double-positive cells (CD4+ CD8+) to CD4+ differentiation of single positive cells-related Treg are needed to suppress the abnormal immune response in autoimmune diseases [32]. The key is that leptin inhibits CD4+CD25+ [33]. When Treg proliferates, Leptin receptor (LEPR) is large. The amount of leptin present on Tregs cell surface means that in vitro leptin-blocking therapy can cause Treg proliferation. In addition, studies have found that, among leptin-deficient mice (ob/ob mice) and LEPR-deficient mice (DB/DB mice), the number of Tregs increases significantly [34, 35]. In terms of actively inhibiting Treg proliferation, the study found that, in LEPR knockout mice, mTOR activity is lower than that of normal mice, accompanied by Treg proliferation [34]. In addition, leptin can inhibit human routine CD4+CD25. Regarding the autophagy process in T cells, the leptin-mTOR axis has emerged as a potential link between immunity and energy state. Also, cells express elongated LEPR on the cell surface, indicating that leptin positively affects B [36].

In contrast, the number of B lymphocytes is reduced. After leptin treatment, the B cell counts increase, leptin is expressed by activating BCL-2, and cyclin D1 is induced into the cell cycle. This promotes B lymphocyte proliferation and inhibits cell cycle apoptosis through JAK-STAT and p38 MAPK-ERK1/2 signals [37]. This also induces pro-inflammatory cytokines (such as Tumor necrosis factor (TNF) and interleukin 6 (IL-6)) and anti-inflammatory and immune regulatory cytokine IL-10 [38].

Furthermore, leptin can increase the number of B cells by promoting proliferation and reducing the rate of apoptosis. It also activates B cells to secrete pro-inflammatory, anti-inflammatory regulatory cell cytokines. Moreover, the formation of B cells in the bone marrow of fasting mice occurs [39].

### Leptin and systemic lupus erythematosus (SLE)

Systemic autoimmune diseases are characterized by inflammatory arthritis and vasculitis. It is interesting to know that, among ob/ob or DB/DB mice, susceptibility to autoimmune diseases is low because leptin appears to play a role in the development of several autoimmune disorders [40]. In fact, both play an important role. SLE can affect all organs and groups related to chronic autoimmune diseases. Many researchers have reported that, among SLE patients compared to healthy people, serum leptin levels are higher, which is associated with SLE disease activity, atherosclerosis, etc [41].

### Pharmacology of leptin

A leptin analog is a hormone approved in the United States to treat congenital leptin deficiency and generalized lipodystrophy. The first leptin analog was Metreleptin, approved in Japan in 2013 [42], in the United States in 2014, and in other countries in 2018 [43]. It is indicated for the treatment of obesity and/or leptin deficiency. Some results have shown that leptin may be used for diabetes and hypertriglyceridemia [44]. For inpatients with DM, Metreleptin may be administered with insulin or insulin sulfonylurea, but dosage adjustment may be necessary to minimize the risk of hypoglycemia [45].

## Discussion

**Figure 2.**
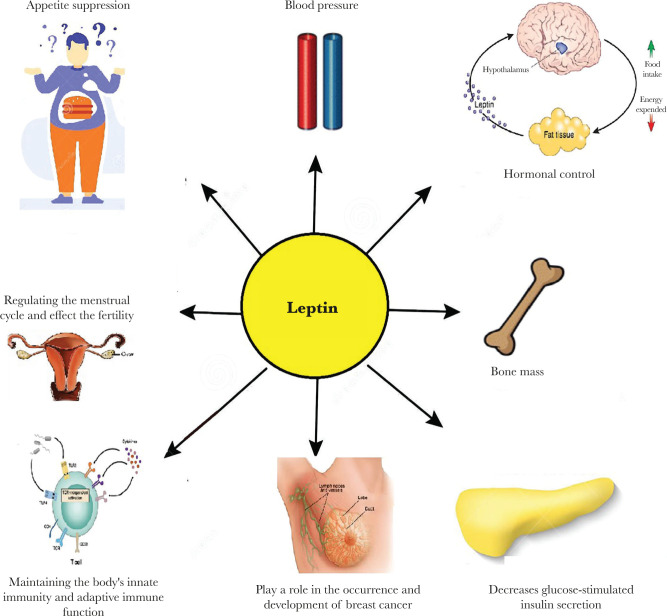
Leptin and its rule to control body function.

The effect of the leptin analog appears to have the same properties in the body: leptin decreases body mass and food intake and suppresses hunger [3]. Also, leptin controls body fat by decreasing triglycerides and decreasing total fat mass as it increases HDL [46]. Also, it appears to have a role in diabetes mellitus (DM) and blood glucose, as leptin analog decreases plasma glucose and plasma insulin increases insulin sensitivity. Some studies have focused on how it treats and restores normal blood glucose levels in DM type 2 [9]. Furthermore, in terms of the reversal and treatment of hypogonadism, immunological changes (as mentioned before), and neurological changes, some studies have shown an increase in gray matter concentration in the anterior cingulate and the medial cerebellum and thalamus in the pulvinar nucleus, increasing the activity of the posterior lobe of the cerebellum [47]. Finally, current research has shown the genetic profile of hepatocellular carcinoma, HepG2, cells [48]. Human leptin may play a role in increasing HepG2 cell proliferation by blocking the ER stress-related apoptotic pathway see (Figure 2) [49].

## Conclusion

The analog of leptin Metreleptin regulates hunger, energy consumption, body mass, blood glucose, lipid metabolism, and levels of lipoproteins in the blood. Moreover, it affects the hypothalamic-pituitary axes (thyroid, adrenal, somatotropic, and gonadotropic), immunity, and brain structure and function. Currently, it has been approved for generalized lipodystrophy, but its effects are potentially valuable for patients with other conditions associated with low, normal, or high levels of leptin serum.

## Acknowledgements

### Conflict of interest

The authors declare that there is no conflict of interest.

### Personal thanks

High gratitude to the collaboration and assistance of everyone in the department of Pharmacology Faculty of the Medicine, University of Baghdad, for their support.

### Authorship

HAAH designed and conducted the research, AHA revised the manuscript and made critical changes, MAN collected and analysed data in order to reduce bias..
